# Surveillance of healthcare-associated infections in Piemonte, Italy: results from a second regional prevalence study

**DOI:** 10.1186/1471-2458-14-558

**Published:** 2014-06-05

**Authors:** Lorena Charrier, Pier Angelo Argentero, Enzo C Farina, Roberto Serra, Francesco Mana, Carla M Zotti

**Affiliations:** 1 Department of Public Health and Paediatrics, University of Torino, Via Santena 5 bis, Torino, Italy; 2 Ospedale di Rivoli, ASL TO3, Via Rivalta 29, Rivoli, Italy; 3 Azienda Ospedaliera Città della Salute e della Scienza, Corso Bramante 88/90, Torino, Italy

**Keywords:** Healthcare-associated infections, Prevalence survey, Risk factors

## Abstract

**Background:**

A prevalence survey of healthcare-associated infections (HAIs) was previously performed in the Piemonte region in 2000. In the decade following the survey, many studies were performed at both the regional and hospital levels, and training courses were developed to address issues highlighted by the survey. In 2010, a second regional prevalence study was performed. The aim of this paper is to present the results of the second prevalence study and discuss them within the context of the HAI prevention and control programmes that have been implemented in the decade since the original survey was conducted.

**Methods:**

The study involved all public hospitals in the Piemonte region. Uni- and multivariate analyses were performed to assess the main risk factors associated with HAIs, including both overall and site-specific infections.

**Results:**

A total of 7841 patients were enrolled: 6.8% were affected by at least one HAI. The highest prevalence of HAIs was found in intensive care units (18.0%, 95% CI 14.0-22.6), while UTIs presented the highest relative frequency (26.7%), followed by respiratory tract infections (21.9%). The age of the patient, hospital size and urinary and central venous catheter status were significantly associated with HAIs.

**Conclusions:**

The study results showed an increase in HAI prevalence, despite prevention and control efforts, as well as training implemented after the first regional survey. Nevertheless, these data are consistent with the current literature. Furthermore, despite its limits, the prevalence approach remains an important means for involving healthcare workers, emphasising HAIs and revealing critical problems that need be addressed.

## Background

In 2000, a region-wide prevalence study of healthcare-associated infections (HAIs) was conducted in the 58 public hospitals (approximately 16,000 beds; 560,000 admissions yearly) of the Piemonte region and in one hospital located in the neighbouring autonomous region of the Valle d’Aosta. The study population was composed of more than 9,000 patients with an HAI prevalence of 4.6% [1]. This study gave us the first, clear overview of HAIs in our region and highlighted the primary obstacles that would be faced in the upcoming years.

Between 2000 and 2010, many efforts were made to support activities aimed at prevention and control of HAIs, and many surveillance studies were performed at both the regional and hospital levels. Based on the results of the surveillance studies as well as the prevalence study, training courses have been offered to the region’s healthcare workers (both medical and nursing) to help eliminate behaviours that are not in line with the existing national and international recommendations (i.e., antibiotic prescription and control measures to be adopted in operating rooms and in clinical care) [1-9].

Between 2006 and 2008, despite the lack of a national system for the surveillance of nosocomial infections, the Italian Centres for Disease Control (CCM) implemented a project (INF-OSS) to coordinate activities aimed at increasing the control of HAIs by implementing standard protocols across all Italian regions and encouraging their involvement in international surveillance systems [10].

In the Piemonte region, over the last decade, programmes have been implemented in all hospitals to establish common surveillance strategies, control and training procedures, and common indicators to evaluate programme efficacy. The programmes were designed to evaluate problems and improvements in the behaviour and performance of healthcare workers, as well as patient outcomes. In this context, the prevalence study represents an effective tool for healthcare workers to identify HAIs and to evaluate the effectiveness of the HAI control programmes.

In 2010, a second regional prevalence surveillance was conducted within the regional programme for HAI prevention and control. The study involved the same 58 public hospitals that participated in the first survey conducted in 2000. These hospitals contained a total of 12,000 regular beds and 429 neonatal beds.

The aim of this paper is to present the results from the second regional prevalence study and to discuss these results in the context of the activities and surveillance implemented within the last decade in the Piemonte region.

## Methods

The survey was conducted between December 2009 and January 2010 within the regional programme for HAI prevention and control. This prevalence study was intended for the surveillance of Healthcare Associated Infections, as required from public hospitals by the Piemonte county government. As the collection of this data was included in monitoring activities mandated by regional law (Circular No.1950/2001 ‘Requisiti di minima per la prevenzione del rischio infettivo nelle strutture ospedaliere della Regione Piemonte’, available on line in the web site of the Italian National Centre for Disease Prevention and Control-CCM: http://www.ccm-network.it/documenti_Ccm/prg_area1/Inf_Oss/Normativa_reg/Piemonte_Prev_minima_strutt_osped_01.pdf) and originates from routine care activities, Ethical Committee consent was not required. Data collection followed the standardised protocol for the HAI prevalence survey arranged within the INF-OSS project, which was performed between 2006 and 2008 and funded by the Italian CCM [10].

### Study population

All 58 public hospitals in the Piemonte region participated in the investigation. The survey included all wards for acute patients, rehabilitation wards, paediatric intensive care units and neonatal care units, but excluded day hospitals, day surgeries and patients under observation in the emergency room or emergency department.

In accordance with the study protocol, all patients in the abovementioned wards at the time of the survey were included in the surveillance study, with the exception of patients transferred from other wards after the start of the survey, patients discharged before the survey began and patients temporarily absent from the ward for examinations or diagnostic procedures who did not come back prior to the end of data collection. Hospitals with more than 500 beds could perform the survey on a sample of patients (50%), randomised by an independent external service to include half of the patients in each ward.

### Data collection

In each hospital, a physician was appointed as the study coordinator. The study coordinator was chosen from among the members of the group responsible for HAI control, and a data collection team was identified and trained. A timetable for the survey was established to identify the day the survey was to be conducted in each ward. Each hospital received the survey protocol and patient forms together with compilation instructions. The items on the patient form were completed with the information obtained from medical and nursing records, temperature charts, data on current therapies, and laboratory and radiology records.

### Data analysis

Patient and infection distributions by hospital type and ward, patient distributions by the presence of medical devices and invasive procedures, and infection distributions according to site are shown as absolute and relative frequencies. The prevalence of infection is shown with corresponding confidence intervals (95% CI).

Univariate and multivariate analyses were conducted to assess the risk of developing HAIs. Using univariate analysis, we evaluated the association between HAIs and each risk factor by calculating the odds ratio (OR) and 95% CI. Parameters found to be statistically significant (p ≤ 0.05) by univariate analysis were inserted into logistic regression models as independent variables, together with gender, age (≤65 vs >65 years), patient provenience (from outpatient clinics or any wards of the same or other hospitals *versus* home) and hospital size (<300 or > 300 beds) to evaluate their independent effects on HAI development, including both overall and site-specific infections (i.e., symptomatic UTIs, respiratory tract infections and bloodstream infections). For surgical site infections (SSIs), we fitted a specific model in which the presence of an SSI was the dependent variable and gender, age, patient provenience, hospital size, the presence of urinary and central venous catheters and index risk were the independent variables. Index risk is a measure used internationally for reporting to stratify SSI data, and it is based on the ASA (American Society of Anesthesiologists) score (3, 4, or 5 vs 1 or 2), wound classification (contaminated or dirty vs clean or clean-contaminated), and procedure duration (>75th percentile). Each risk factor represents 1 point; thus, the SSI risk index ranges from 0 (no risk factors present) to 3 (greatest risk).

For all tests, the significance level was set at α = 0.05.

All analyses were performed using STATA 12 software.

## Results

### Population and infections

A total of 7841 patients were surveyed (47.6% men, 50.4% women, 2% missing data).

The average enrolment rate was 63.2% (minimum 21%, maximum 100%) of available bed-sites; the 3 hospitals with more than 500 bed-sites adhered to the study protocol and randomised the bed-sites for surveillance.

Age was determined for 98.5% of the patients; the mean age of the population under surveillance was 64.5 years (SD = 22.1). Excluding paediatric patients (326 < 14 years old), 25% of the patients were <58 years old, 50% were <72 years old and 75% were <81 years old.

In total, 1204 patients (15.4%) had community infections not related to their current hospitalisation. A total of 531 HAIs were diagnosed, with an overall prevalence of 6.77% (95% CI 6.2-7.3). Including the 49 patients with two sites of infection, there were 580 HAIs overall.

Table 1 describes the population studied and the number, relative frequency and prevalence of HAIs overall, by hospital type (primary, secondary, tertiary) and by ward.

**Table 1:** Population surveillance data, number, relative frequency and prevalence of healthcare-associated infections (overall and according to site) according to hospital type and ward

	**No. of patients (%)**	**No. of HAIs (%) HAIs Prevalence % (95% CI)**	**No. of UTIs (prevalence %)**	**No. of BSIs (prevalence %)**	**No. of RTIs (prevalence %)**	**No. of SSIs (prevalence %)**	**No. of other sites (prevalence %)**
**Hospital type**							
Primary	2217 (28.3)	132 (24.8) 6 (5.0-7.0)	47 (2.1)	11 (0.5)	29 (1.3)	11 (0.5)	34 (1.5)
Secondary	3760 (47.9)	261 (49.1) 7 (6.1-7.8)	74 (2.0)	27 (0.7)	72 (1.9)	30 (0.8)	58 (1.5)
Tertiary	1864 (23.8)	138 (26.0) 7.4 (6.3-8.7)	34 (1.8)	17 (0.9)	26 (1.4)	16 (0.8)	45 (2.4)
*Total*	*7841*	*531 (100) 6.8 (6.2-7.3)*	*155 (2.0)*	*55 (0.7)*	*127 (1.6)*	*57 (0.7)*	*137 (1.7)*
**Ward**							
General medicine	1779 (22.7)	109 (20.7) 6.1 (5.0-7.2)	42 (2.4)	15 (0.8)	27 (1.5)	5 (0.3)	20 (1.1)
General surgery	801 (10.2)	43 (8.2) 5.4 (3.8-6.9)	5 (0.6)	2 (0.2)	3 (0.4	18 (2.2)	15 (1.9)
Medical specialties	2647 (33.8)	208 (39.5) 7.9 (6.9-8.9)	73 (2.7)	19 (0.7)	60 (2.3)	12 (0.4)	44 (1.7)
Surgical specialties	1980 (25.3)	92 (17.5) 4.6 (3.8-5.7)	24 (1.2)	5 (0.2)	9 (0.5)	19 (0.9)	35 (1.8)
Intensive care units	333 (4.26)	60 (11.4) 18.0 (14.0-22.6)	9 (2.7)	10 (3.0)	26 (7.8)	3 (0.9)	12 (3.6)
Paediatrics	285 (3.64)	15 (2.8) 5.3 (3.0-8.5)	2 (0.7)	4 (1.4)	-	-	9 (3.2)
*Total*	7825* (100)	*527° (100) 6.7 (6.2-7.3)*	*155 (2.0)*	*55 (0.7)*	*125° (1.6)*	*57 (0.7)*	*135° (1.7)*

*When stratification by ward was performed, we observed missing data for 16 patients.

°When stratification by ward was performed, we observed missing data for 4 infections.

**Figure 1:**
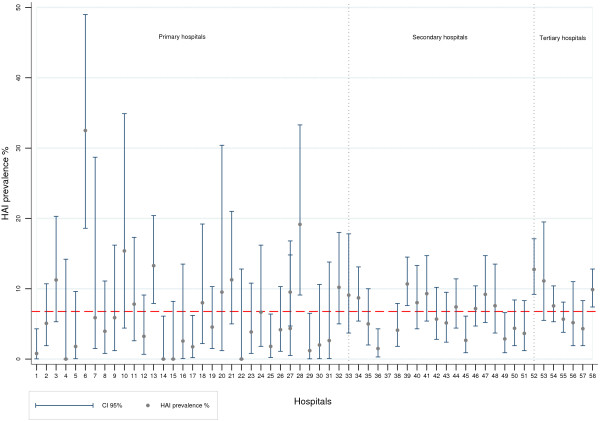
**Prevalence (%) and 95% CI of HAIs in the 58 participating hospitals and according to hospital type (hospitals 1 to 33 are “primary” hospitals, 34 to 52 are “secondary hospitals”, 53 to 58 are “tertiary” hospitals).** The dashed line represents the mean regional HAI prevalence (6.77%).

Altogether, 285 of 7825 patients (3.6%) were admitted to paediatric wards, and 41 patients of paediatric age were admitted into adult wards. Among patients in paediatric wards, the HAI prevalence was 5.3% (Table 1), whereas the HAI prevalence among all patients of paediatric age was 6.4% (95% CI 3.8-9.1).Figure 1 represents the individual HAI prevalence per participating hospital and according to hospital type. It shows the great variability in HAI prevalence, especially when comparing the primary hospital group to the secondary and tertiary hospitals, which produce more precise estimates.

Table 2 shows the prevalence of patients exposed to a medical device or a surgical treatment: 24.3% of patients were not exposed to devices or experienced other invasive procedures; 38.1% experienced two exposures, and 12.9% experienced more than two.

**Table 2:** Frequency of medical device placement or invasive procedures

**Devices/Invasive procedures**	**No. of patients**	**Prevalence %**	**Missing data %**
Surgical procedures during the stay	2159	28.1	1.9
Urinary catheter within 7 days prior to the study	2461	31.7	0.9
Urinary catheter at the time of admission	881	11.3	0.4
**On the day of the study, presence of:**			
Urinary catheter	2155	27.5	0.1
Peripheral venous catheter (CVP)	4107	52.6	0.3
Central venous catheter (CVC)	1106	14.1	0.2
Mechanical ventilation	226	2.9	0.3
Parenteral nutrition	608	7.8	0.2

Table 3 shows the sites of infection, with the type of microorganism found at the site of the HAIs. The microorganism prevalence was calculated for 524 bacterial cultures; the most commonly found microorganisms were Enterobacteriaceae (especially *Escherichia coli*) and Gram-positive bacteria (especially *Staphylococcus aureus).* We performed antimicrobial resistance evaluation and observed the following: 59% of cases were *Staphylococcus aureus* oxacillin-resistant strains*;* there were no ampicillin and/or *glycopeptide-*resistant *Enterococci;* 30% of cases were *ESBL*-producing *Enterobacteriaceae;* 66% of cases were *Pseudomonas aeruginosa* non-multi-drug-resistant strains, but 29.5% were carbapenemase-producingand 9% were carbapenems-resistant *Pseudomonas aeruginosa*; and 20% of cases were carbapenem-resistant *Acinetobacter baumannii.*

**Table 3:** Healthcare-associated infections and the site of infection: number, prevalence rate, relative frequency and prevalence of cultured microorganisms

	**HAIs (No)**	**Prevalence% (95% CI)**	**Relative frequency %**	**Microorganism (%)**
Surgical site infection	57	0.73 (0.54-0.92)	9.83	*Escherichia coli (29.4)*
				*Staph. aureus (23.5)*
				*Pseudomonas aeruginosa (8.8)*
Urinary tract infection (symptomatic)	155	1.98 (1.67-2.28)	26.7	*Escherichia coli (42.8)*
				*Enterococcus faecalis (9.9)*
				*Pseudomonas aeruginosa (6.6)*
				*Proteus mirabilis (6.6)*
				*Candida albicans (5.9)*
Bloodstream infection	55	0.70 (0.52-0.87)	9.48	*Staph. epidermidis (20.4)*
				*Staph. aureus (18.5)*
				*Escherichia coli (18.5)*
Respiratory tract infection	127	1.62 (1.34-1.90)	21.9	*Staph. aureus (23.4)*
				*Pseudomonas aeruginosa (20.8)*
				*Escherichia coli (10.4)*
Other sites^	186*	2.44 (2.11-2.80)	32.1	
*Total*	*580*	*7.40 (6.83-7.99)*	*100*	

^Gastrointestinal infections, skin and connective tissue infections, cardiovascular infections, ear infections, other UTI, systemic infections.

*75 missing and 2 ‘unknown’ are included.

### Risk factor analysis

Using univariate analysis, the relationship between the main risk factors (medical devices/invasive procedures) and the presence of HAIs was evaluated:

– 9.3% of 226 patients with mechanical ventilation on the day of the study had a respiratory tract infection (RTI) vs 1.4% of patients who were not mechanically ventilated (OR = 7.24, 95% CI 4.43-11.83)

– The presence of an intravenous catheter was a risk factor for bloodstream infections (BSIs) (OR = 5.73, 95% CI 2.80-14.40), but if we consider peripheral and central venous catheterisation separately, only the latter was significantly related to BSI (OR = 7.46, 95% CI 4.36-12.78)

– In the symptomatic UTI group, 155 patients (1.98%) had infections at the time of the study. In 73.2% of the patients with symptomatic UTIs, a urinary catheter was present during the seven days preceding the study vs 30.8% of the patients without this infection (OR 6.13; 95% CI 4.26 - 8.82)

– For 2054 of 2159 surgical operations, data pertaining emergency treatment or elective surgery were available; we did not find a significant association between treatment in emergency situations and the development of SSIs (OR = 1.38, 95% CI 0.74-2.59)

– A total of 2054 patients out of 2159 who underwent surgical treatment were classified by surgical wound type: 1132 patients was classified as “clean” and had 15 SSIs (1.33%); 660 were “clean-contaminated” with 12 infections (1.82%); 188 were “contaminated” with 14 infections (7.45%), and 74 were “dirty infected” with 3 infections (4.05%). The surgical wound class was significantly related to the presence of SSIs (p < 0.001); specifically, the risk of SSI was 2.42 higher for surgical wounds classified as “clean-contaminated”, “contaminated” or “dirty infected” versus the “clean” category (95% CI 1.29-4.54)

– Calculation of the Risk Index was possible for only 1626 surgical treatments out of 2159 because only these procedures had records available for all of the necessary parameters (ASA score, wound class, duration of operation). In these cases, the presence of SSIs was found to be statistically associated with the Risk Index (p = 0.003).

We did not find a significant relationship between hospital type, length of stay (number of days between hospital admission and data collection), case mix (proxy of complexity of healthcare delivery) of the hospitals involved and the presence of HAIs. On the other hand, the hospital size (more vs less than 300 beds) was found statistically associated to HAIs in the univariate analysis: OR = 1.39 (CI95%: 1.17-1.66).

The parameters found to be statistically significant (p ≤ 0.05) in the univariate analysis were subsequently inserted into logistic regression models (multivariate analyses) together with age, gender and patience provenience, to evaluate their independent effect in determining HAIs, both overall and according to site (odds ratios and corresponding confidence intervals are shown in Table 4). Logistic regression analyses showed that age was the risk factor associated with the most infections, along with hospital size and the presence of urinary catheter or central venous catheter. Mechanical ventilation was only associated with RTIs, while parenteral nutrition was not a significant risk factor for HAIs.

**Table 4:** Results from the logistic regression analyses: HAIs (overall and according to site) and associated risk factor assessment

	**All HAIs**		**symptomatic UTIs**		**BSIs**		**RTIs**	
	**OR (95% CI)**	**p**	**OR (95% CI)**	**p**	**OR (95% CI)**	**p**	**OR (95% CI)**	**p**
**Age (>65)**	**1.35 (1.09-1.67)**	**0.006**	**2.35 (1.48-3.73)**	**<0.001**	0.70 (0.39-1.24)	0.226	**1.64 (1.06-2.54)**	**0.026**
**Female**	0.99 (0.82-1.21)	0.938	**1.76 (1.23-2.52)**	**0.002**	0.94 (0.54-1.67)	0.851	**0.55 (0.37-0.83)**	**0.004**
**Hospital size (>300 beds)**	**1.34 (1.09-1.63)**	**0.004**	1.23 (0.86-1.75)	0.252	**2.13 (1.18-3.85)**	**0.012**	**1.52 (1.03-2.26)**	**0.037**
**Patient provenience^**	**1.92 (1.54-2.39)**	**<0.001**	1.42 (0.95-2.13)	0.088	1.59 (0.84-3.02)	0.157	**3.01 (2.01-4.51)**	**<0.001**
**Urinary catheter**	**2.44 (1.99-3.00)**	**<0.001**	**5.28 (3.53-7.90)**	**<0.001**	1.26 (0.67-2.33)	0.471	**1.76 (1.15-2.69)**	**0.009**
**CVC**	**2.29 (1.79-2.92)**	**<0.001**	1.34 (0.87-2.15)	0.176	**4.92 (2.54-9.55)**	**<0.001**	**2.40 (1.49-3.85)**	**<0.001**
**Mechanical ventilation**	1.17 (0.76-1.78)	0.474	0.73 (0.28-1.87)	0.509	1.68 (0.66-4.32)	0.278	**2.17 (1.16-4.04)**	**0.015**
**Parenteral nutrition**	1.28 (0.95-1.72)	0.102	0.93 (0.52-1.65)	0.797	1.05 (0.49-2.25)	0.902	1.60 (0.95-2.67)	0.080

^From the same hospital (ICU or other wards) or LTF vs. “home”.

Results in bold indicate statistically significant associations (p<0.05).

The model fitted for SSIs showed that the index risk was the only risk factor significantly associated (p = 0.007) with the development of SSIs (data not shown).

## Discussion

The current prevalence study was performed 10 years after the original study in the same hospitals. The results showed an increase in HAI prevalence (6.8% vs 4.6%), despite efforts made in prevention, control and training within the last decade. However, this information must not be interpreted negatively, as our data are consistent with studies conducted in similar epidemiological settings (Liguria [11], Veneto [12]), and with studies conducted in Italy and in Europe in both recent and past years [13-21]. Within our region in the last ten years, we have implemented many programmes to strengthen the management, surveillance, control and training for HAIs; these programmes were implemented in all hospitals enrolled in the study. Moreover, prevalence studies are only a useful tool for understanding the frequency of HAIs if these studies are periodically repeated in the same context, and in the past ten years, the management of care has undergone major changes. For example, the case mix (especially surgical) was enhanced (data from the Piemonte region showed an increase in case mix from 2000 to 2010 in all facilities with surgical specialist activities and in more than 1/3 of the hospitals involved in the study), and medical devices and invasive procedures were improved in comparison to the previous regional prevalence study (e.g., the percentage of patients with intravenous or urinary catheters on the day of surveillance increased from 62.3% to 63.8% and from 19.9% to 27.5%, respectively; CVCs were used in 5.3% of patients in general medicine and in 13.6% in general surgery in 2000 vs 12% and 50% in 2010, respectively).

Risk factor analysis confirmed a high frequency of predisposing conditions, and some risk factors, such as mechanical ventilation and urinary and venous catheterisations, were confirmed to be critical in the management of good clinical practices. Additionally, a previous stay in another hospital/ward or in the intensive care unit increased the patients’ risk for HAIs and must be carefully considered, especially in tertiary hospitals that admit more difficult cases and critical patients from other hospitals or from long-term facilities.

Moreover, although a prevalence study is not the best tool for surveying SSIs, this study exposed the difficulties in obtaining complete data regarding surgical operations, which are useful in measuring and accurately estimating risk. Furthermore, despite its limitations, this prevalence study also enabled us to obtain a quick picture of antibiotic resistance in our hospitals.

## Conclusions

Due to changes in management of care and patient complexity, prevalence studies are not ideal for analysing trends in HAI frequency. However, descriptive studies remain a useful means for evaluating area hospitals and help to elucidate major problems with patient care by drawing more attention to and increasing proficiency in healthcare workers.

Specifically, in our region, all public hospitals have been involved in the survey, so the results can be useful for comparing hospital performance, especially among facilities with similar complexity.

## Competing interests

The authors declare that they have no competing interests.

## Authors’ contributions

LC: statistical analysis, drafting of the manuscript. PAA: study design (medical area), critical revision of the manuscript. ECF: study design (surgical area), critical revision of the manuscript. RS: study design (microbiological area). FM: technical support in data collection. CMZ: obtained funding, study concept and design, study supervision, drafting of the manuscript. Piemonte Collaborative Group for Healthcare-associated infections: coordination of data collection at local level. All authors read and approved the final manuscript.

## Pre-publication history

The pre-publication history for this paper can be accessed here:

http://www.biomedcentral.com/1471-2458/14/558/prepub
